# The Tale of Stridor and Wheezing in an Infant

**DOI:** 10.1155/2021/8847436

**Published:** 2021-02-09

**Authors:** Umer Muhammad, Marcus Shaffer, John Bishara

**Affiliations:** Women and Children's Hospital, CAMC/West Virginia University-Charleston, Charleston, WV, USA

## Abstract

Recurrent respiratory papillomatosis is a respiratory disease caused by human papillomavirus and can infect any part of the aerodigestive tract, but the larynx is most involved (Derkay et al. 2010). This report is a discussion about a 7-month-old male that presented to our institution for respiratory distress. He was admitted to the Pediatric Intensive Care Unit (PICU) for stabilization, observation, and further treatment and management due to an acute RSV infection. Initial efforts failed to improve his respiratory failure. A bronchoscopy was performed and showed various flesh-colored lesions throughout the larynx, vocal cords, and tracheal tree just above the carina. Pediatric otolaryngology performed an emergent debulking surgery to alleviate his respiratory failure. He has had multiple exacerbations of his condition since then and has required frequent debulking procedures with a few trials of intralesional bevacizumab therapies.

## 1. Introduction

Recurrent respiratory papillomatosis (RRP) is a result of an infection caused by the human papilloma virus (HPV) [1]. The virus causes abnormal proliferation of the normal squamous epithelium leading to complications such as airway obstruction, mucus plug formation, and airway compromise, which may recur over time. In the pediatric population, it is typically diagnosed around the age of 2 to 3 years but earlier onset of the disease may be attributed to infections caused by more aggressive genotypes of HPV, vertical transmission during birth, or transplacental infection acquired from an infected mother [2].

Pathology into HPV DNA subtype analysis helps determine the HPV genotype such as 6 and 11 vs. 16, 18, and 31 [3]. This is essential since HPV 16 has been associated with the more aggressive forms of papillomatosis with possible risk for distal airway involvement and malignant transformation [4]. Involvement of extralaryngeal sites such as the trachea, bronchial epithelium, and mucous glands usually occurs in addition with upper airway involvement and is also more common in children vs. adults, occurring in 3–15% of cases [5, 6]. The overall risk of malignant transformation is low in children (<1%), since majority of infections are caused by HPV genotypes 6 and 11. However, genotype analysis helps predict the recurrence and aggressiveness of the disease process [3]. Histopathology may reveal koilocytes (HPV-infected dysplastic squamous cells with a “raisinlike” nucleus).

Tracheostomy is usually avoided as it can increase the risk of recurrence and development of lower airway papillomas from distal spread. But, the need for a tracheostomy may vary based upon individual case circumstances such as upper airway obstruction [3].

## 2. Case Presentation

A 7-month-old male with a history of NICU admission for neonatal asbstinence syndrome for 3 weeks after in utero drug and tobacco exposure (opioids, benzodiazepines, and marijuana) was transferred from an outlying institution due to respiratory distress, hypoxemia, and stridor. He received oxygen via mask delivery, dexamethasone, and several racemic epinephrine treatments prior to his transfer for stabilization. However, his respiratory distress did not improve with the above interventions.

Upon presentation to the emergency room, he had worsening respiratory distress. He was started on oxygen supplementation after a trial of nebulization treatment. However, he progressed into respiratory failure. A viral panel was positive for RSV. Hisrespiratory failure worsened, and he required endotracheal intubation with invasive mechanical ventilation. Although his respiratory status improved, he continued to have intermittent episodes of hypoxemia. A chest X-ray showed multifocal opacities typical of a RSV infection. He had a similar episode of respiratory failure two months prior that required endotracheal intubation.

His intermittent respiratory failure with worsening hypoxemia while on invasive mechanical ventilation prompted an evaluation by pediatric pulmonology. It was decided that the patient have a more thorough airway evaluation via flexible bronchoscopy.

## 3. Diagnostic Focus and Assessment

**Figure 1:**
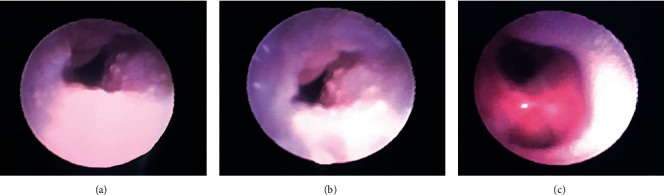
Images collected during flexible bronchoscopy. (a) Lesions protruding from the tracheal wall mucosal. (b) Narrowing of the trachea secondary to multiple lesions. (c) Normal-appearing area ∼3 cm above the carina.

During flexible bronchoscopy, widespread, sessile, 3–5 mm mucosal lesions resembling verruca were noted throughout the larynx, vocal cords, and trachea approximately 3 cm above the carina; images recorded are shown in Figure 1. However, the procedure was aborted after the patient experienced multiple severe hypoxemic episodes due to tracheal obstruction from these lesions. Pediatric otolaryngology was consulted for surgical management. An emergent debulking surgery was performed.

## 4. Therapeutic Focus and Assessment

**Figure 2:**
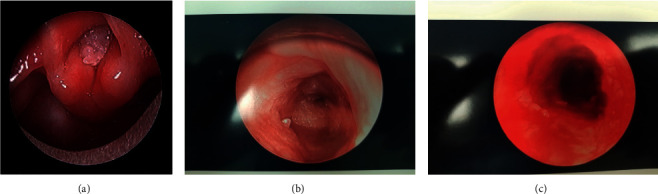
Images collected s/p debulking surgery via rigid bronchoscopy. (a) Lesions obscuring the vocal cord(s) [7]. (b) Lesions in the distal trachea. (c) Tracheal lumen.

The debulking surgery was performed using a microdebrider blade. Many lesions were excised, and images were obtained for preoperative and postoperative comparison (Figure 2). The patient tolerated the procedure well, and his respiratory distress improved significantly in the postoperative period leading to extubation shortly thereafter.

## 5. Follow-Up and Outcome

He was discharged home with follow-up for planned debulking procedures. Unfortunately, he presented several more times forrespiratory failure, each requiring intubation and bronchoscopic evaluation of his airway with debulking surgeries. Due to the recurrence of papillomatosis obstructing the vocal cords, a tracheostomy was performed. The patient has had a total of four debulking surgeries from January 2020 to March 2020. Multimodality treatment with intralesional vs. systemic administration of bevacizumab has also been considered as an adjunctive measure to minimize the number of debulking surgeries and to potentially increase the time interval between subsequent procedures. However, the patient had only a modest response to similar treatments in the past. Due to disease progression, the patient has developed bilateral true vocal cord fixation and subglottic stenosis, recently requiring a laryngoplasty approach for debulking procedures.

## 6. Discussion

RRP is the most common cause of benign laryngeal neoplasm in children [1]. The disease process may affect any part of the aerodigestive tract; the larynx is the most involved anatomical location [1]. The lesions are usually multiple, stemlike protrusion or “cauliflowerlike” in appearance and may bleed with manipulation. Suspected tracheobronchial involvement- or recurrence-related complications are further investigated with imaging of the chest and bronchoscopy. Chest X-ray is usually normal unless there is lung parenchymal involvement. The CT scan may show walled cysts with nodules, atelectasis, and bronchiectasis with mucus plugging [2]. Airway visualization (i.e., flexible or rigid bronchoscopy) remains superior, as it may aid with direct visualization of lesions and biopsies for further analysis.

The recurrent nature of the disease course warrants repeated surgical procedures, and the mean number of surgeries required can be as high as 4.4 procedures per year [8]. Consequently, this exposes patients to various complications such as anesthesia-related complications, damage to healthy tissue, tracheal stenosis, perforation, and socioeconomical burdens on families [9].

In addition, adjunct treatments with alpha-interferon and antiviral medications such as cidofovir have shown to be effective as well. These agents may increase the length of the time intervals between surgeries or decrease the total number of surgeries that the patients may require [9]. Vascular endothelial growth factor (VEGF) inhibitor, such as bevacizumab, is another feasible choice for adjunct treatment therapies for recurrent respiratory papillomatosis [10, 11]. However, these agents are prone to limitations due to dosing recommendations, systemic vs. intralesional mode of administration, and minimal response to therapies [7, 12]. HPV vaccinations may also be an effective measure to prevent the overall risk of disease transmission in the pediatric population as majority of these infections are vertically transmitted from a high-risk mother [9].

## 7. Conclusion

Recurrent respiratory papillomatosis infections in children are typically acquired during vertical transmission at birth or transplacental infections. The risk factors for recurrence, aggressive nature of the disease with distal spread, and malignant potential include firstborn child, vaginal delivery, women with a history of HPV infection (subtype 16), and history of prior invasive interventions. However, among infants born to women with such a history, the risk of RRP is <1% [4]. The prognosis of disease also varies based upon the risk factors mentioned above with less aggressive types of infections having a good outcome and more aggressive types of infections requiring frequent debulking surgeries and tracheostomy [8].

## Data Availability

The academic and clinical percentile data used to support and discuss the findings of this case report are included within the article.
